# Notes and News

**Published:** 1891-11-07

**Authors:** 


### Notes and News.

Collected and Communicated.

Messrs. Arnold and Sons, the well-known instrument makers, have found it necessary to extend their premises and to acquire additional accommodation for their various departments. They have secured the lease of 31, West Smithfield, and of 2, Giltspur Street, E.C. Their business in future will, we are informed, be carried as regards its various departments at Nos. 31, 35, 36, and 37, West Smithfield, and at 2, Giltspur Street, E.C. The retail business will be conducted at 35, West Smithfield, and the orthopædic branch at 2, Giltspur Street.

We paid a visit to the Clapham Maternity Hospital last week. The institution has been open now two years for the reception of in-patients, and has made marked progress during that time. During the present year 105 cases have been admitted. No maternal deaths have occurred either amongst the in or out patients. Indeed, since the commencement of the work, which originated in an out-patients' department only, 1,800 cases have been attended without the occurrence of a fatal case. This reflects great credit on the ladies engaged in the work. The institution opens its doors to unmarried girls for the purpose of assisting them to return to a respectable life, but is otherwise a purely medical institution, engaged in the training of gentlewomen in midwifery and monthly nursing. There is a Resident House Surgeon. The students live with the doctor in her private house. The students have been 24 in number during the present year. The wards contain from three to four beds in each. They are furnished with rigid simplicity, but are light and airy. They are vacated in rotation for the purpose of disinfection. The floors are washed frequently with permanganate of potash, which stains them to a dark brown colour, by no means a disagreeable contrast to the pale green painting of the walls. From a sanitary point of view this method has great advantages. The nursing is superintended by a fully trained Matron, whose services are voluntary. The utmost care is taken to keep the establishment within its means, and the institution has in consequence a balance in hand. The work, however, is capable of great development were funds forthcoming. The institution derives its income from patients' payments—of varying amounts—and subscriptions and donations. There are two dispensaries for women and children in connection with the hospital at Stockwell and Wandsworth. These are deservedly popular, and all three institutions are doing good work.

The Dublin hospitals participating in municipal grants are subjected to an annual inspection. This year the Special Committee report favourably of the Jervis Street Hospital, the St. Vincent's, the Mater Misericordæ, Sir Patrick Dunn's Hospital, Dr. Stevens' Hospital, the Cork Street Fever Hospital, the National Lying-in Hospital, and, in fact, of most of the city hospitals. At the Mercer's and the City of Dublin Hospital they had a few complaints to make on the subject of cleanliness in one or two departments.

It is attracting notice that cases of lead poisoning are of no infrequent occurence in Leeds. Dr. Robert Stephenson, of that town, deposed that in the six weeks previous to September he had had six such cases, and that not a week had passed during the last two years without bringing him one. It is significant that these instances have all been confined to the district served by the Rural Sanitary Authority. The general opinion is that the Local Government Board should hold an inquiry into the whole question.

The ceremony of laying the memorial stone and the chief corner stone of the extension of the Morley Convalescent Home at Dover for London working men was performed last week by the Lord Mayor of London and Mr. Arnold Morley, M.P., respectively. The home was established in 1883, and now has accommodation for thirty-six patients. Since it was opened by Mr. Samuel Morley in that year 2,700 London working men have benefited by residence there. The extension which has formed the object of the ceremonial will provide fifty additional beds, so that in all there will be accommodation for eighty-two patients when the work is completed.

The cry for a Pasteur Institution in India is again raised. Deaths from hydrophobia in India are not few in number, and cases of it frequently occur among the British soldiers and the Anglo-Indian community. Only recently the Commander-in-Chief was compelled to order the transmission of an unhappy soldier, showing symptoms of hydrophobia, to Paris to be treated by the French scientist at the cost of the State, although there is no regulation which empowers the military authorities to take such a step in the case of a sick soldier. It is known that since the success achieved by M. Pasteur, several Anglo-Indians bitten by mad dogs had to be sent direct to Paris from India to be admitted into the Pasteur Hospital. It is thus apparent that the establishment of a Pasteur Institute in India will be a boon, especially to the British forces and the Anglo-Indian community. For the present one Pasteur Hospital in a central spot, say Nagpore, will meet the public requirements.

The Salford Royal Hospital may almost be said to have reached a crisis in its history, for there is a financial deficit amounting to £4,863 on the last four years. An annual expenditure of a thousand pounds over income is not a pleasant thing for the management to contemplate, and of course cannot be maintained indefinitely. If a town, like Wigan, thanks to a system of carefully superintended monthly collections from the employés of the various local industries, can raise over £3,000 a year for the funds of its hospital, surely some similar plan might be devised and carried out in Salford. As many as 18,718 cases were treated in the Hospital, and at the patients' homes, in the course of last year, and with such evidence of the usefulness of the institution one can hardly think that the people of Salford will allow the work of the out and home patients' department to be reduced, but this must evidently be the result if they do ot support the hospital liberally.

